# Supplementary material for: Associations of Body Mass Index with Ventilation Management and Clinical Outcomes in Invasively Ventilated Patients with ARDS Related to COVID-19—Insights from the PRoVENT-COVID Study
Source: J Clin Med. 2021 Mar 11;10(6):1176. doi: 10.3390/jcm10061176 (PMC8000207; doi:10.3390/jcm10061176)
Supplement: Supplementary file 1 [file jcm-10-01176-s001.pdf]

SUPPLEMENTARY MATERIAL

Figure S1 - Cumulative Distribution Plots of Ventilatory Variables at the Second Day of Ventilation

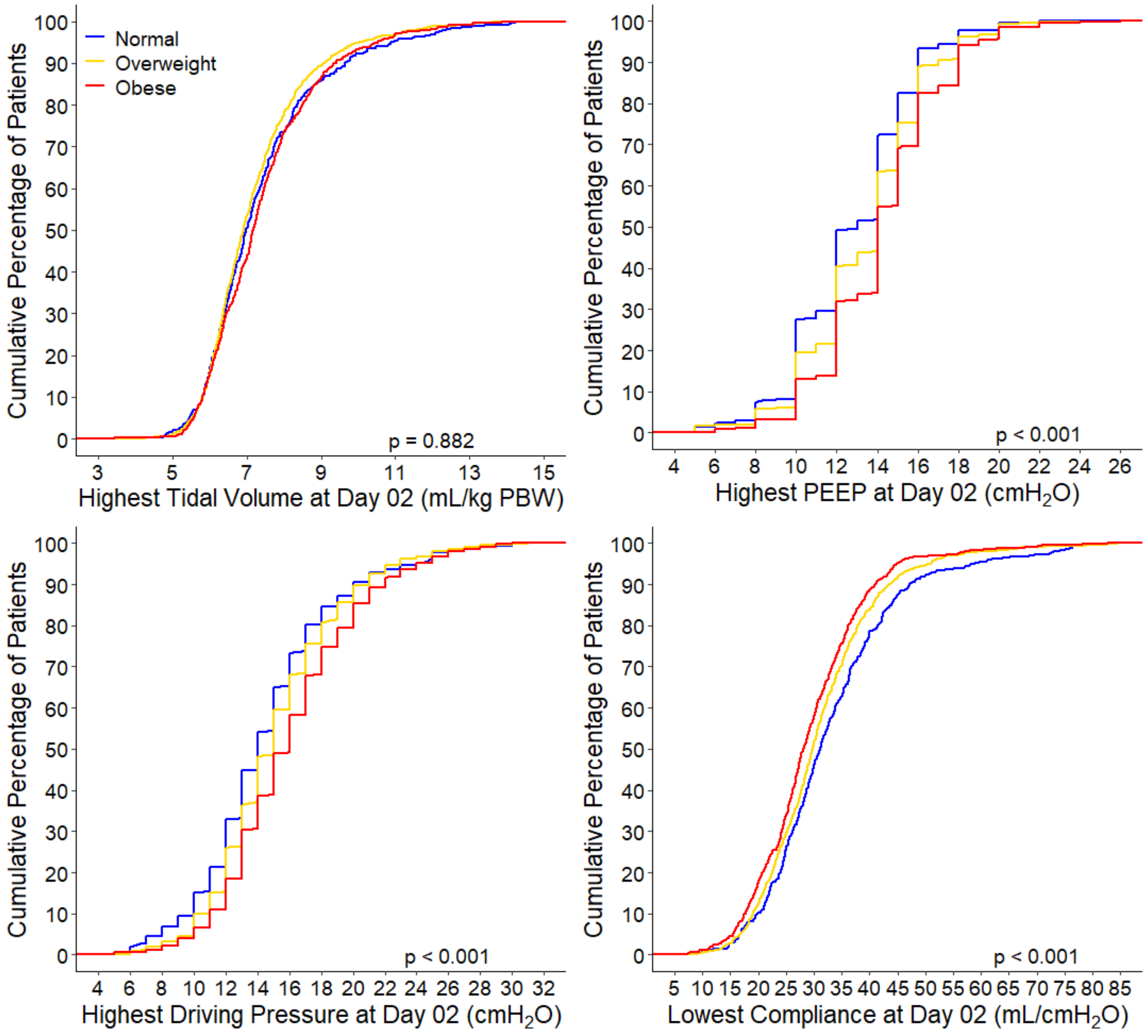

Curves that rise more slowly represents higher values in the studied variables. Highest or lowest values were extracted from three measurements available in the first day of ventilation. *P* values calculated from Kruskal–Wallis tests.

Figure S2 - Cumulative Distribution Plots of Ventilatory Variables at the Third Day of Ventilation

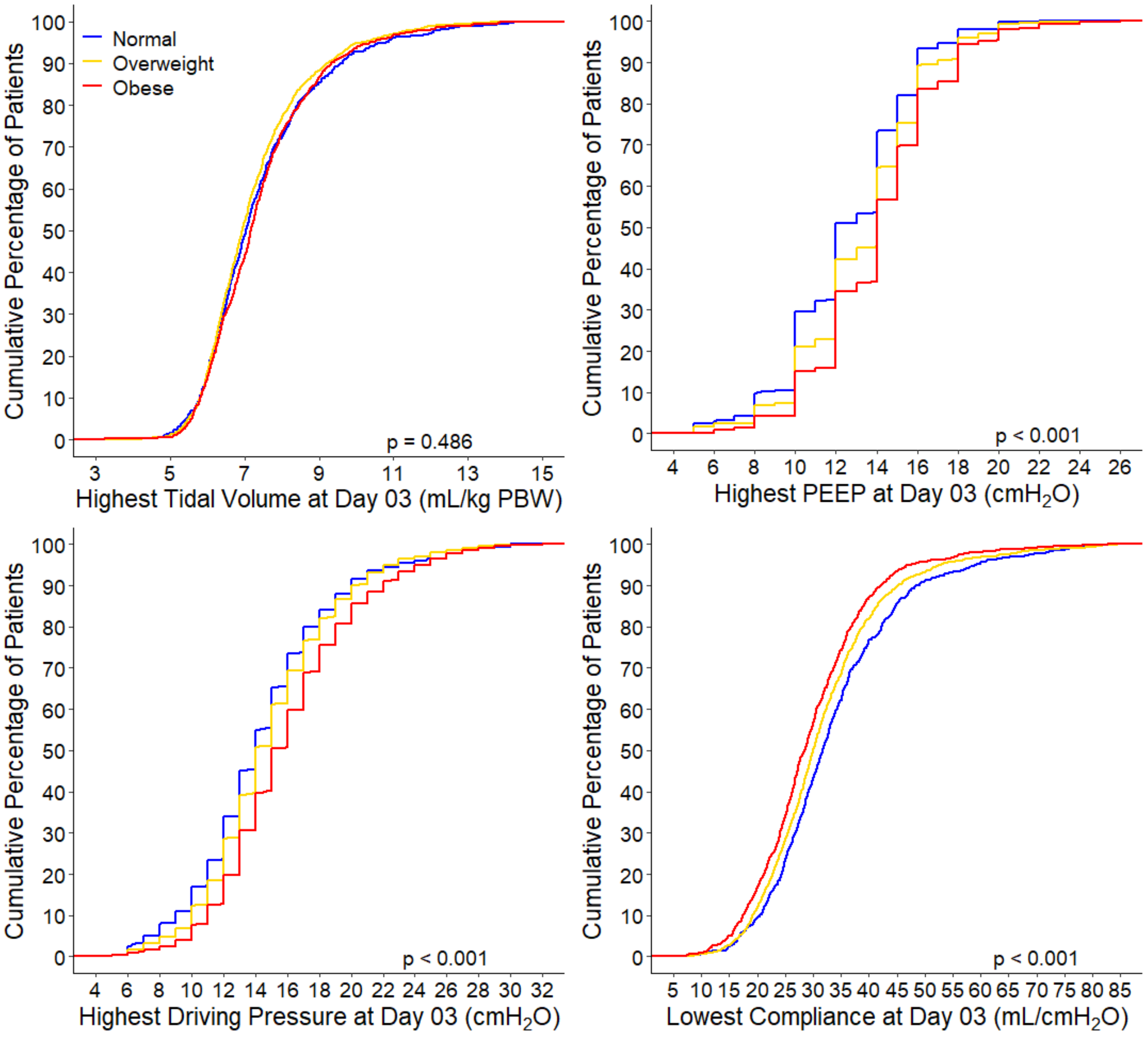

Curves that rise more slowly represents higher values in the studied variables. Highest or lowest values were extracted from three measurements available in the second day of ventilation. *P* values calculated from Kruskal–Wallis tests.

Figure S3 - Cumulative Distribution Plots of Ventilatory Variables at the Fourth Day of Ventilation

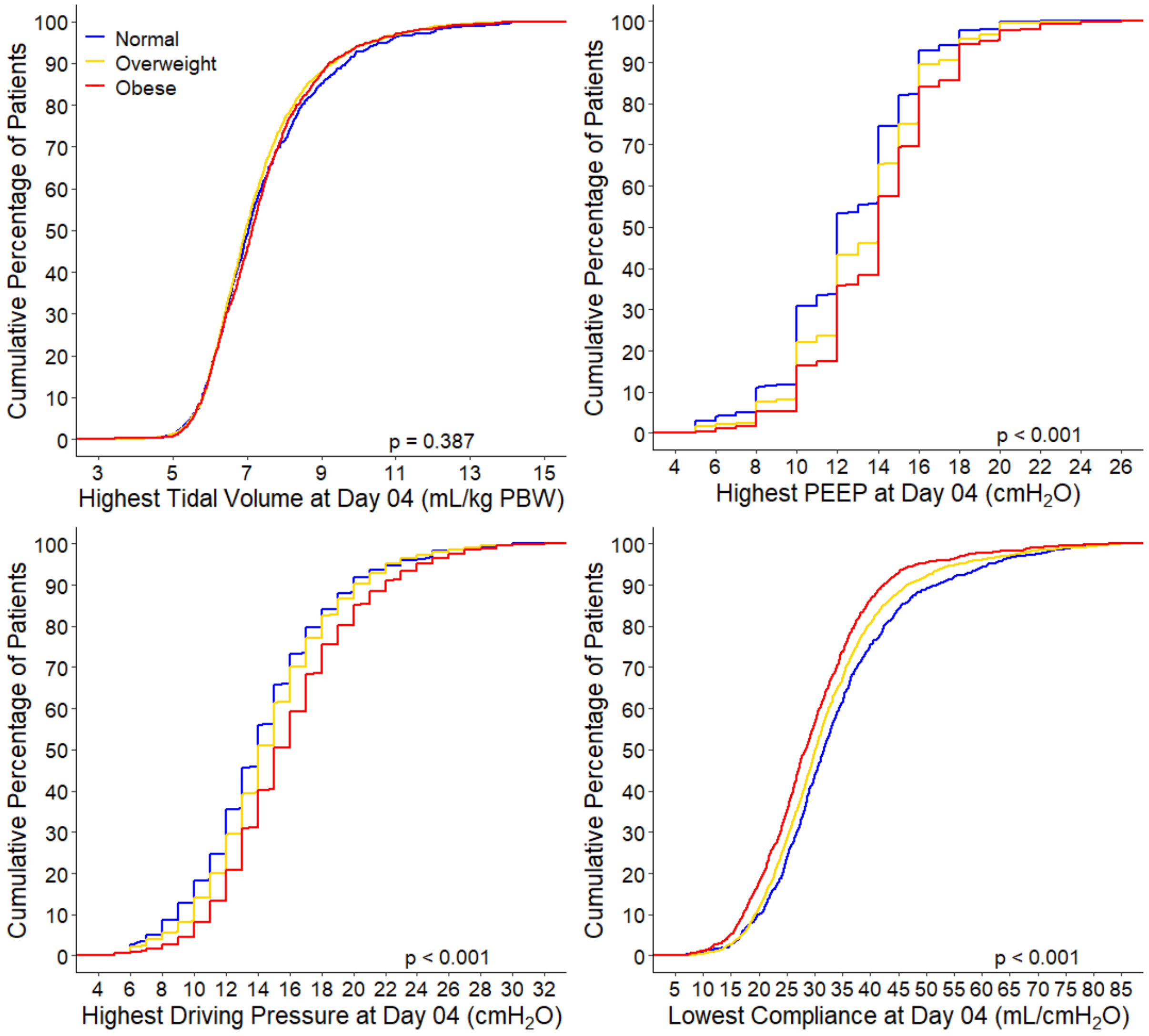

Curves that rise more slowly represents higher values in the studied variables. Highest or lowest values were extracted from three measurements available in the third day of ventilation. *P* values calculated from Kruskal–Wallis tests.

Figure S4 - Ventilatory Variables and Oxygenation in the First Four Days of Ventilation

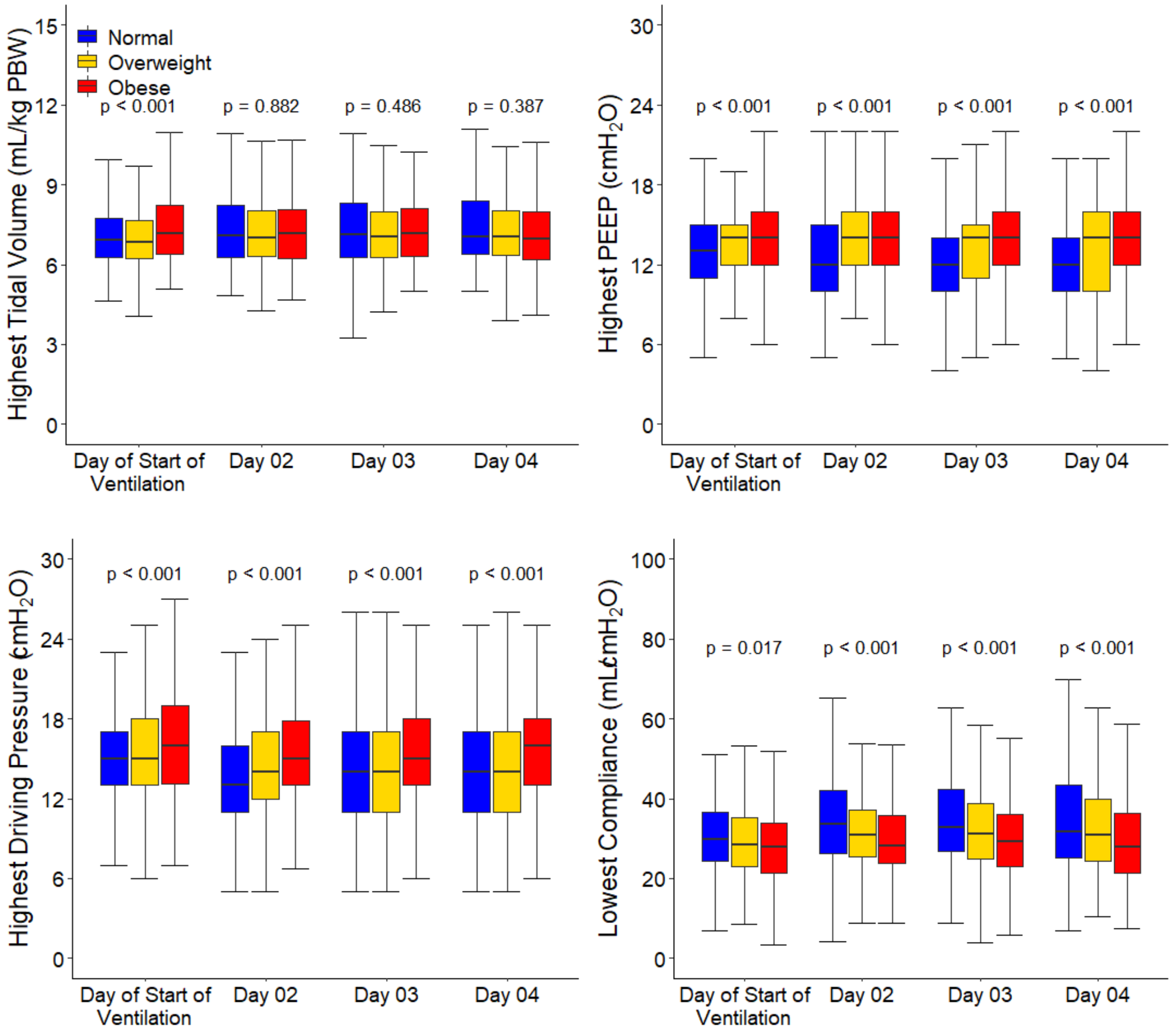

Boxes represent median and interquartile range. Whiskers extend 1.5 times the interquartile range beyond the first and third quartiles per the conventional Tukey method. Highest or lowest values were extracted from three or four measurements available in each day of ventilation. *P* values calculated from Kruskal–Wallis tests.

Figure S5 - Kaplan-Meier Curve for 90-Day Mortality in the Overall Population and According to ARDS Severity

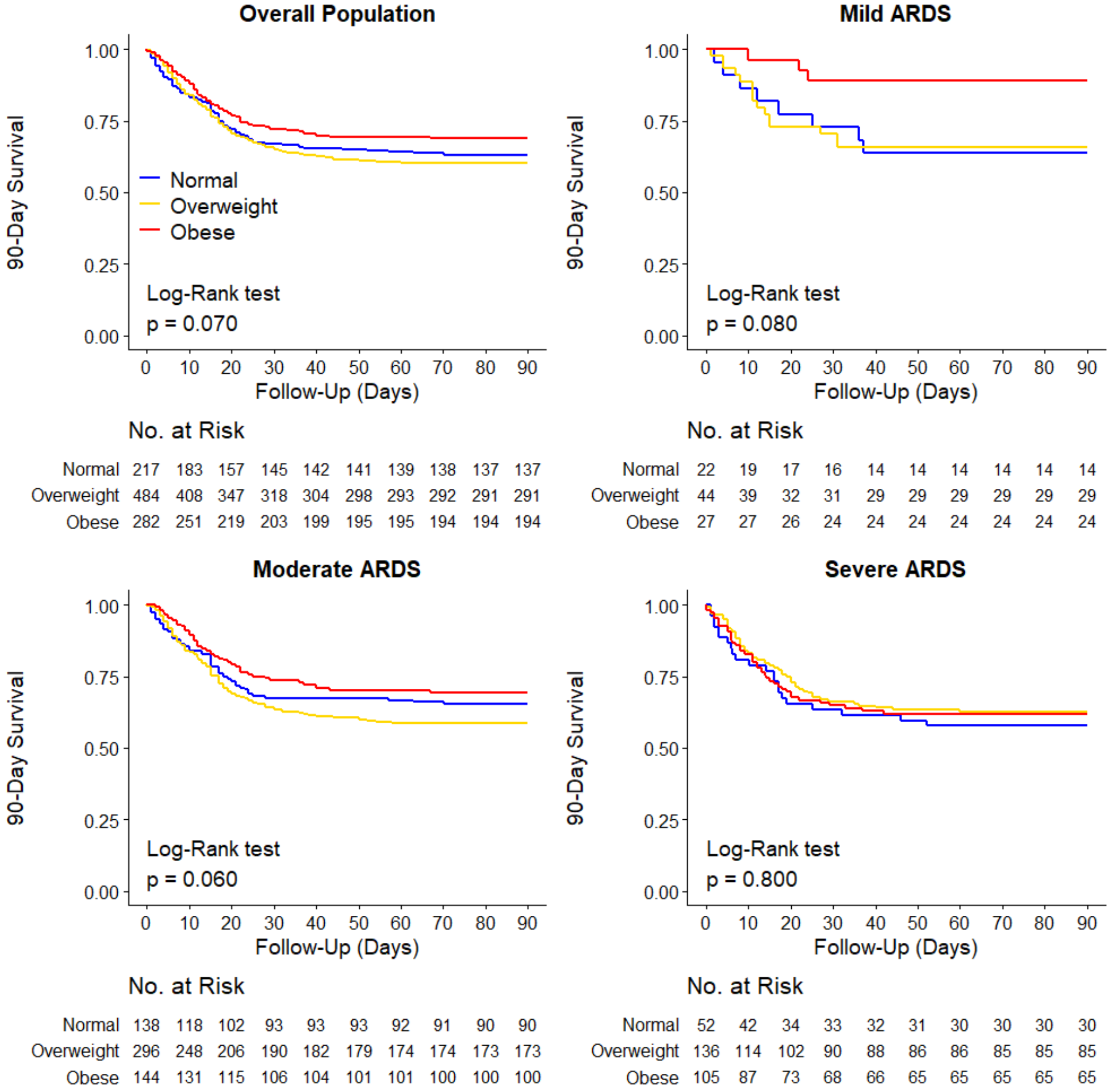

P values calculated from Log-Rank test. Unadjusted and adjusted (shared-frailty) Cox proportional hazard models are shown in eTables 4 and 5.

Figure S6 - Cumulative Distribution Plots of Ventilatory Variables and Oxygenation at the First Day of Ventilation

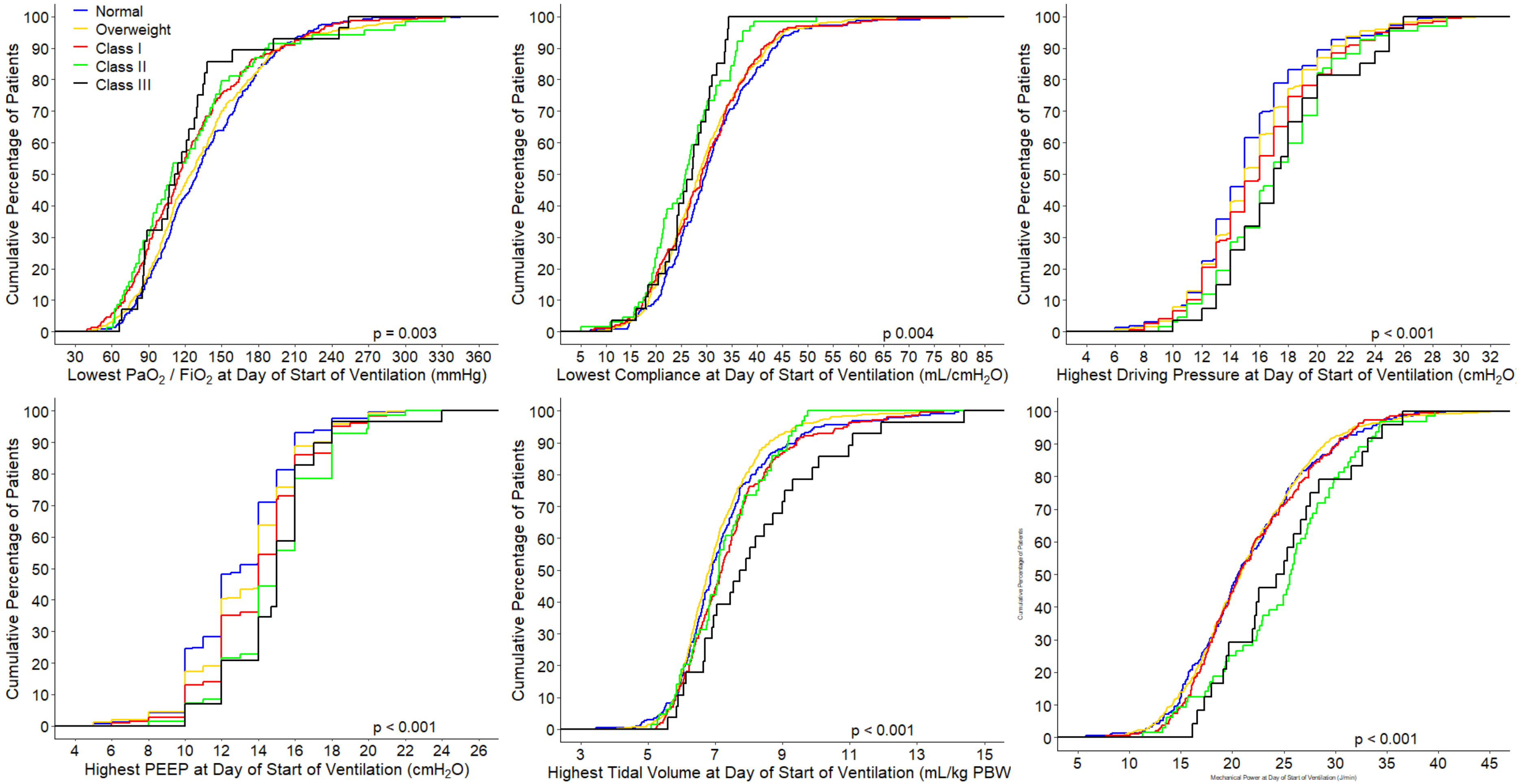

Curves that rise more slowly represents higher values in the studied variables. *P* values calculated from Kruskal-Wallis tests. Highest or lowest values were extracted from three measurements available in the third day of ventilation.

Figure S7 - Kaplan-Meier Curve for 28-Day Mortality in the Overall Population and According to ARDS Severity

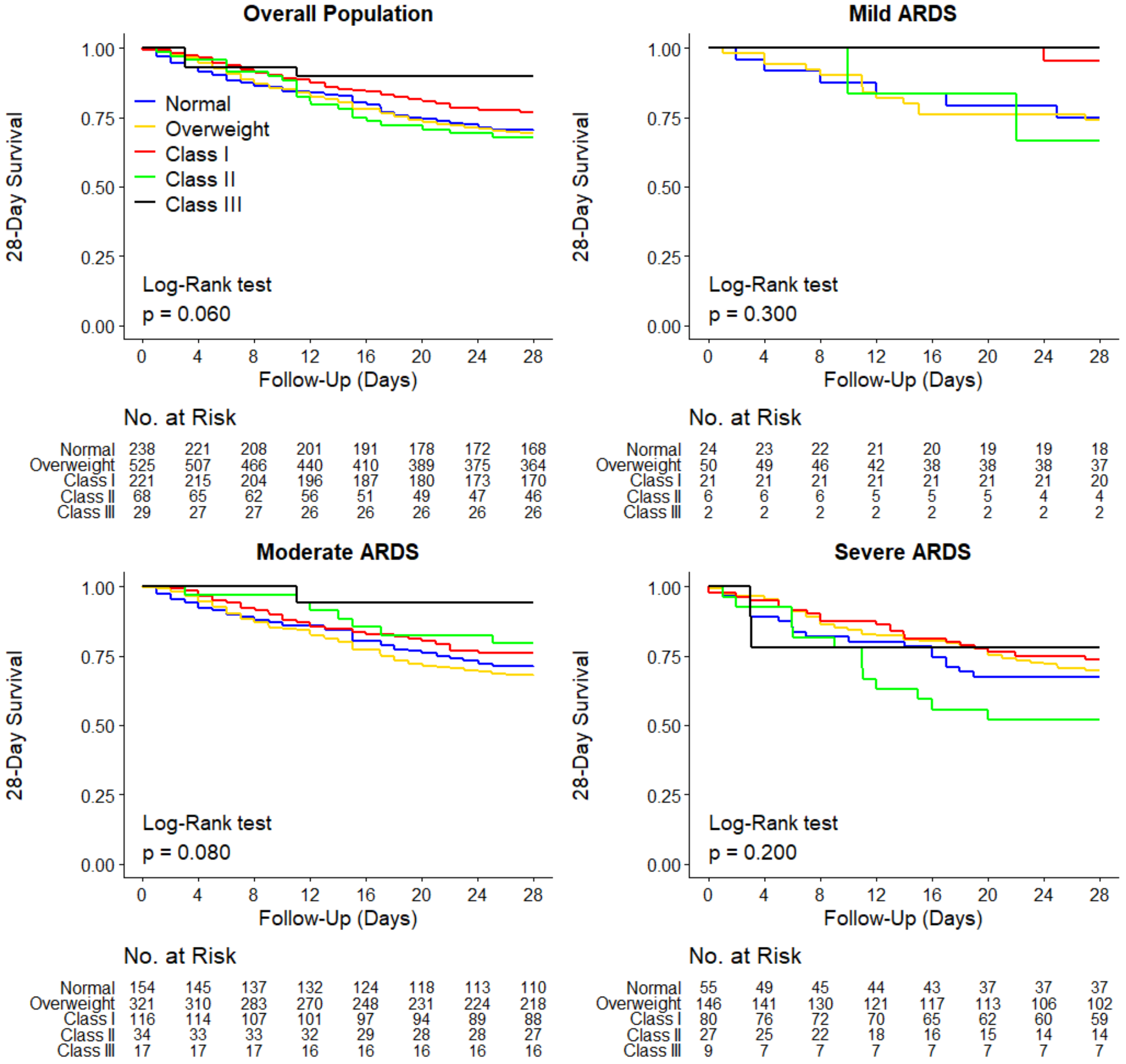

P values calculated from Log-Rank test. Unadjusted and adjusted (shared-frailty) Cox proportional hazard models are shown in eTable 8.

Table S1. - Univariable Assessment of Factors Associated with 28-Day Mortality.

	Hazard Ratio (95% CI)	*p* value
**Body mass index category**		
Normal	1 (Reference)	
Overweight	1.02 (0.77 to 1.35)	0.890
Obese	0.78 (0.57 to 1.09)	0.140
**Demographic characteristics**		
Age	2.02 (1.74 to 2.35)	< 0.001
Male gender	1.19 (0.91 to 1.55)	0.210
**Severity of ARDS**		
Mild	1 (Reference)	
Moderate	1.38 (0.88 to 2.16)	0.155
Severe	1.46 (0.91 to 2.35)	0.113
**Co-existing disorders**		
Hypertension	1.58 (1.26 to 1.98)	< 0.001
Heart failure	1.73 (1.09 to 2.74)	0.019
Diabetes	1.65 (1.29 to 2.11)	< 0.001
Chronic kidney disease	1.27 (0.76 to 2.10)	0.360
Chronic obstructive pulmonary disease	1.61 (1.12 to 2.31)	0.010
Active hematological neoplasia	1.52 (0.67 to 3.44)	0.320
Active solid neoplasia	1.72 (0.93 to 3.19)	0.082
**Previous medication**		
Angiotensin converting enzyme inhibitor	1.39 (1.05 to 1.83)	0.021
Angiotensin II receptor blocker	1.35 (0.98 to 1.87)	0.069
**Vital signs at the day of start of ventilation**		
Heart rate	1.17 (1.04 to 1.30)	0.008
Mean arterial pressure	0.80 (0.71 to 0.91)	< 0.001
**Laboratory tests at the day of start of ventilation**		
Creatinine	1.06 (0.99 to 1.14)	0.087
pH	0.66 (0.59 to 0.73)	< 0.001
**Ventilation support at the day of start of ventilation**		
Tidal volume per PBW	1.05 (0.96 to 1.15)	0.310
PEEP	1.19 (1.06 to 1.35)	0.004
Compliance	1.00 (0.88 to 1.14)	0.970
**Organ support at the day of start of ventilation**		
Fluid balance	1.18 (1.06 to 1.32)	0.003
Use of vasopressor	1.35 (1.01 to 1.80)	0.044
**Rescue therapy at the day of start of ventilation**		
Prone positioning	0.71 (0.55 to 0.93)	0.014
Use of NMBA	0.95 (0.73 to 1.23)	0.680

Continuous variables were included after standardization and the hazard ratio represents the increase in one standard deviation of the variable.

Variables with a $p < 0.200$ were selected for inclusion in the multivariable model.

Table S2 - Multivariable Assessment of Factors Associated with 28-Day Mortality

	Hazard Ratio (95% CI)	*p* value
**Body mass index category**		
Normal	1 (Reference)	
Overweight	1.10 (0.83 to 1.47)	0.500
Obese	0.89 (0.63 to 1.25)	0.510
**Demographic characteristics**		
Age	1.82 (1.55 to 2.14)	< 0.001
**Co-existing disorders**		
Hypertension	1.28 (1.00 to 1.64)	0.048
Heart failure	1.25 (0.78 to 2.02)	0.350
Diabetes	1.36 (1.04 to 1.76)	0.023
Chronic obstructive pulmonary disease	1.53 (1.06 to 2.22)	0.025
**Previous medication**		
Angiotensin converting enzyme inhibitor	0.98 (0.72 to 1.32)	0.880
**Vital signs at the day of start of ventilation**		
Heart rate	1.15 (1.02 to 1.30)	0.023
Mean arterial pressure	0.88 (0.78 to 0.99)	0.034
**Laboratory tests at the day of start of ventilation**		
pH	0.71 (0.62 to 0.81)	< 0.001
**Ventilation support at the day of start of ventilation**		
PEEP	1.25 (1.10 to 1.42)	< 0.001
**Organ support at the day of start of ventilation**		
Fluid balance	1.08 (0.96 to 1.21)	0.210
Use of vasopressor	1.09 (0.80 to 1.48)	0.580
**Rescue therapy at the day of start of ventilation**		
Prone positioning	0.59 (0.44 to 0.77)	< 0.001

Continuous variables were included after standardization and the hazard ratio represents the increase in one standard deviation of the variable.

Table S3 - Adjusted Effect of Body Mass Index Categories in 28-Day, ICU, Hospital and 90-Day Mortality*

	Mild ARDS		Moderate ARDS		Severe ARDS	
	Effect Estimate (95% CI)	*p* value	Effect Estimate (95% CI)	*p* value	Effect Estimate (95% CI)	*p* value
**28-Day mortality**						
Body mass index category						
Normal	1 (Reference)		1 (Reference)		1 (Reference)	
Overweight	HR, 0.60 (0.17 to 2.12)	0.430	HR, 1.11 (0.77 to 1.59)	0.580	HR, 0.94 (0.53 to 1.69)	0.850
Obese	HR, 0.23 (0.04 to 1.38)	0.110	HR, 0.72 (0.45 to 1.14)	0.170	HR, 1.04 (0.55 to 1.98)	0.900
**ICU mortality**						
Body mass index category						
Normal	1 (Reference)		1 (Reference)		1 (Reference)	
Overweight	OR, 0.65 (0.15 to 2.86)	0.573	OR, 1.68 (1.05 to 2.70)	0.030	OR, 0.85 (0.40 to 1.81)	0.671
Obese	OR, 0.17 (0.02 to 1.42)	0.102	OR, 1.04 (0.58 to 1.86)	0.895	OR, 0.89 (0.39 to 2.03)	0.786
**Hospital mortality**						
Body mass index category						
Normal	1 (Reference)		1 (Reference)		1 (Reference)	
Overweight	OR, 0.48 (0.11 to 2.01)	0.315	OR, 1.68 (1.04 to 2.73)	0.033	OR, 0.92 (0.43 to 1.98)	0.835
Obese	OR, 0.11 (0.01 to 0.89)	0.038	OR, 1.05 (0.58 to 1.92)	0.858	OR, 1.03 (0.45 to 2.38)	0.939
**90-Day mortality**						
Body mass index category						
Normal	1 (Reference)		1 (Reference)		1 (Reference)	
Overweight	HR, 0.57 (0.19 to 1.73)	0.320	HR, 1.19 (0.84 to 1.68)	0.330	HR, 0.93 (0.54 to 1.59)	0.790
Obese	HR, 0.22 (0.04 to 1.19)	0.079	HR, 0.87 (0.56 to 1.35)	0.540	HR, 1.04 (0.58 to 1.86)	0.900

Continuous variables were included after standardization and the hazard ratio represents the increase in one standard deviation of the variable.

* All models adjusted for age, hypertension, heart failure, diabetes, chronic obstructive pulmonary disease, use of angiotensin converting enzyme inhibitor, heart rate, mean arterial pressure, pH, PEEP, fluid balance, use of vasopressor and prone positioning on the day of start of ventilation.

Table S4 - Baseline Characteristics of the Included Patients According to BMI Category

	Normal (*n* = 244)	Overweight (*n* = 531)	Class I (*n* = 225)	Class II (*n* = 70)	Class III (*n* = 29)	*p* value
Age, years	67.0 (60.0 - 73.0)	66.0 (59.0 - 73.0)	63.0 (54.0 - 71.0)	59.0 (49.2 - 66.8)	55.0 (48.0 - 59.0)	< 0.001
Male gender – no (%)	186 (76.2)	407 (76.6)	153 (68.0)	44 (62.9)	12 (41.4)	< 0.001
Body mass index, kg/m^2	23.9 (22.9 - 24.6)	27.3 (26.2 - 28.5)	31.8 (30.9 - 33.2)	37.0 (35.9 - 38.2)	42.6 (41.5 - 46.1)	< 0.001
Transferred under invasive ventilation	51 (20.9)	82 (15.4)	36 (16.0)	17 (24.3)	6 (20.7)	0.163
Days between intubation and admission	0.0 (0.0 - 0.0)	0.0 (0.0 - 0.0)	0.0 (0.0 - 0.0)	0.0 (0.0 - 0.0)	0.0 (0.0 - 0.0)	0.492
Use of non-invasive ventilation – no (%)	15 (6.8)	45 (9.4)	16 (7.9)	6 (9.1)	2 (7.7)	0.824
Duration of non-invasive ventilation, hours	5.5 (2.0 - 48.0)	8.0 (2.0 - 15.1)	8.0 (2.8 - 20.5)	7.0 (4.0 - 7.8)	4.5 (2.8 - 6.2)	0.797
Chest CT scan performed – no (%)	83 (35.8)	169 (33.2)	72 (33.0)	21 (30.9)	10 (35.7)	0.932
Lung parenchyma affected – no (%)						0.712
0%	3 (3.6)	6 (3.5)	4 (5.6)	0 (0.0)	1 (10.0)	
25%	32 (38.1)	51 (29.7)	24 (33.3)	6 (28.6)	3 (30.0)	
50%	26 (31.0)	52 (30.2)	21 (29.2)	6 (28.6)	1 (10.0)	
75%	20 (23.8)	53 (30.8)	19 (26.4)	7 (33.3)	3 (30.0)	
100%	3 (3.6)	10 (5.8)	4 (5.6)	2 (9.5)	2 (20.0)	
Chest X-ray performed – no (%)	127 (85.2)	289 (86.5)	121 (84.6)	38 (88.4)	16 (88.9)	0.967
Quadrants affected – no (%)						0.380
1	14 (11.1)	21 (7.2)	5 (4.2)	2 (5.4)	0 (0.0)	
2	24 (19.0)	67 (23.0)	35 (29.2)	9 (24.3)	4 (25.0)	
3	37 (29.4)	72 (24.7)	35 (29.2)	12 (32.4)	8 (50.0)	
4	51 (40.5)	131 (45.0)	45 (37.5)	14 (37.8)	4 (25.0)	
Severity of ARDS – no (%)						0.651
Mild	24 (10.0)	51 (9.8)	21 (9.5)	6 (8.7)	2 (7.1)	
Moderate	157 (65.7)	326 (62.3)	119 (53.8)	35 (50.7)	17 (60.7)	
Severe	58 (24.3)	146 (27.9)	81 (36.7)	28 (40.6)	9 (32.1)	
Co-existing disorders – no (%)						
Hypertension	74 (30.3)	186 (35.0)	80 (35.6)	26 (37.1)	8 (27.6)	0.599
Heart failure	9 (3.7)	31 (5.8)	6 (2.7)	1 (1.4)	1 (3.4)	0.231
Diabetes	41 (16.8)	115 (21.7)	59 (26.2)	20 (28.6)	11 (37.9)	0.016
Chronic kidney disease	8 (3.3)	31 (5.8)	8 (3.6)	0 (0.0)	0 (0.0)	0.092
Baseline creatinine, µmol/L*	77.0 (61.0 - 98.0)	78.0 (64.0 - 97.0)	78.0 (60.0 - 99.0)	73.0 (66.0 - 95.8)	71.0 (61.5 - 82.0)	0.794

Table S4 - Baseline Characteristics of the Included Patients According to BMI Category

	Normal (*n* = 244)	Overweight (*n* = 531)	Class I (*n* = 225)	Class II (*n* = 70)	Class III (*n* = 29)	*p* value
Liver cirrhosis	0 (0.0)	2 (0.4)	0 (0.0)	1 (1.4)	0 (0.0)	0.324
Chronic obstructive pulmonary disease	20 (8.2)	41 (7.7)	17 (7.6)	5 (7.1)	2 (6.9)	0.998
Active hematological neoplasia	5 (2.0)	10 (1.9)	1 (0.4)	0 (0.0)	0 (0.0)	0.469
Active solid neoplasia	6 (2.5)	14 (2.6)	5 (2.2)	2 (2.9)	0 (0.0)	0.995
Neuromuscular disease	2 (0.8)	3 (0.6)	2 (0.9)	1 (1.4)	0 (0.0)	0.632
Immunosuppression	9 (3.7)	8 (1.5)	5 (2.2)	2 (2.9)	0 (0.0)	0.337
Previous medication – no (%)						
Systemic steroids	10 (4.1)	17 (3.2)	7 (3.1)	3 (4.3)	1 (3.4)	0.897
Inhalation steroids	21 (8.6)	58 (10.9)	23 (10.2)	9 (12.9)	13 (44.8)	< 0.001
Angiotensin converting enzyme inhibitor	33 (13.5)	93 (17.5)	45 (20.0)	12 (17.1)	3 (10.3)	0.357
Angiotensin II receptor blocker	24 (9.8)	57 (10.7)	27 (12.0)	13 (18.6)	4 (13.8)	0.319
Beta-blockers	40 (16.4)	98 (18.5)	54 (24.0)	13 (18.6)	4 (13.8)	0.280
Insulin	14 (5.7)	38 (7.2)	14 (6.2)	7 (10.0)	5 (17.2)	0.183
Metformin	29 (11.9)	77 (14.5)	42 (18.7)	15 (21.4)	8 (27.6)	0.044
Statins	70 (28.7)	155 (29.2)	77 (34.2)	15 (21.4)	8 (27.6)	0.322
Calcium channel blockers	47 (19.3)	79 (14.9)	42 (18.7)	15 (21.4)	10 (34.5)	0.048
Vital signs at the day of start of ventilation						
Heart rate, bpm**	84.0 (71.5 - 97.0)	84.0 (73.0 - 97.1)	85.0 (76.5 - 98.0)	90.5 (79.6 - 96.8)	80.5 (76.0 - 97.8)	0.201
Mean arterial pressure, mmHg**	78.7 (73.0 - 86.0)	80.0 (73.5 - 87.5)	82.0 (76.3 - 90.0)	81.2 (74.2 - 88.8)	83.8 (77.5 - 89.0)	0.007
Laboratory tests at the day of start of ventilation						
pH**	7.36 (7.30 - 7.41)	7.37 (7.32 - 7.41)	7.37 (7.31 - 7.41)	7.35 (7.30 - 7.40)	7.35 (7.31 - 7.39)	0.389
Worst PaO_2 / FiO_2, mmHg***	130.0 (101.0 - 166.9)	125.0 (98.5 - 162.4)	115.5 (88.1 - 148.8)	108.8 (83.3 - 145.0)	113.1 (87.2 - 131.3)	0.004
$PaCO_2$, mmHg**	44.5 (39.5 - 51.3)	44.3 (38.8 - 49.6)	44.4 (39.5 - 49.5)	45.1 (41.8 - 53.1)	46.9 (39.8 - 56.6)	0.169
Lactate mmol/L**	1.2 (0.9 - 1.5)	1.2 (1.0 - 1.4)	1.1 (0.9 - 1.4)	1.0 (0.8 - 1.3)	1.2 (1.0 - 1.4)	0.082
Organ support at the day of start of ventilation – no (%)						
Continuous sedation	231 (95.1)	506 (95.5)	218 (96.9)	69 (98.6)	29 (100.0)	0.577
Inotropic or vasopressor	192 (79.0)	412 (77.7)	175 (77.8)	47 (67.1)	19 (65.5)	0.151
Vasopressor	192 (79.0)	412 (77.7)	174 (77.3)	47 (67.1)	19 (65.5)	0.154
Inotropic	18 (7.4)	17 (3.2)	8 (3.6)	2 (2.9)	0 (0.0)	0.088
Fluid balance, mL	696.7 (29.0 - 1441.0)	515.8 (7.3 - 1239.3)	499.0 (-19.9 - 1321.0)	365.0 (-11.0 - 1477.0)	377.0 (6.0 - 887.0)	0.456

Table S4 - Baseline Characteristics of the Included Patients According to BMI Category

	Normal (*n* = 244)	Overweight (*n* = 531)	Class I (*n* = 225)	Class II (*n* = 70)	Class III (*n* = 29)	*p* value
Urine output, mL	692.5 (333.8 - 1116.2)	647.5 (350.0 - 1145.0)	732.5 (445.0 - 1115.0)	600.0 (350.0 - 1090.0)	567.5 (387.5 - 1207.5)	0.669
Ventilation support at the day of start of ventilation						
Assisted ventilation – no (%)	73 (30.0)	161 (30.6)	65 (28.9)	13 (18.6)	6 (20.7)	0.251
Tidal volume, mL/kg PBW**	6.4 (5.9 - 6.9)	6.4 (5.9 - 7.0)	6.6 (5.9 - 7.5)	6.5 (5.9 - 7.3)	7.0 (6.4 - 8.2)	< 0.001
PEEP, cmH_2O**	12.0 (10.0 - 14.0)	12.7 (11.0 - 14.5)	13.5 (11.3 - 15.0)	14.0 (12.0 - 15.0)	14.7 (13.5 - 15.5)	< 0.001
Peak pressure, cmH_2O**	25.2 (22.8 - 28.9)	26.6 (23.5 - 29.3)	27.0 (24.7 - 30.4)	28.8 (26.7 - 32.0)	30.0 (27.5 - 34.1)	< 0.001
Driving pressure, cmH_2O**	13.0 (11.2 - 15.3)	13.7 (12.0 - 16.0)	14.2 (12.0 - 16.7)	15.2 (13.9 - 18.0)	15.5 (13.5 - 18.0)	< 0.001
Mechanical power, J/min**	18.1 (14.7 - 21.6)	18.2 (15.3 - 21.9)	18.0 (15.5 - 22.6)	21.8 (17.6 - 25.3)	22.3 (18.6 - 26.8)	< 0.001
Compliance, mL/cmH_2O**	36.2 (28.7 - 45.1)	33.4 (26.8 - 41.1)	33.1 (26.5 - 39.5)	30.6 (24.0 - 34.0)	30.6 (25.6 - 33.7)	< 0.001
Total respiratory rate, mpm**	21.7 (19.3 - 24.0)	21.7 (19.8 - 24.0)	21.3 (19.0 - 23.7)	22.5 (20.0 - 25.0)	24.0 (22.0 - 26.0)	0.001
FiO_2**	0.54 (0.45 - 0.65)	0.57 (0.47 - 0.66)	0.60 (0.52 - 0.70)	0.64 (0.51 - 0.72)	0.65 (0.54 - 0.75)	< 0.001
$etCO_2$, mmHg**	35.7 (32.0 - 40.7)	36.5 (32.4 - 41.6)	37.5 (33.6 - 43.3)	39.4 (35.4 - 44.3)	41.4 (35.4 - 45.0)	< 0.001
Rescue therapy at the day of start of ventilation – no (%)						
Prone positioning	61 (25.3)	161 (30.8)	68 (30.9)	27 (39.7)	9 (31.0)	0.209
Duration, hours[a]	9.0 (6.0 - 14.0)	8.0 (4.0 - 13.5)	8.0 (3.5 - 12.5)	6.0 (2.1 - 14.1)	10.0 (4.0 - 14.0)	0.270
Recruitment maneuver	3 (1.5)	12 (2.8)	3 (1.6)	1 (1.8)	1 (3.7)	0.699
ECMO	1 (0.4)	0 (0.0)	3 (1.4)	0 (0.0)	0 (0.0)	0.081
Use of NMBA	54 (22.2)	154 (29.1)	56 (24.9)	23 (32.9)	10 (34.5)	0.151
Hours of use of use[a]	0.0 (0.0 - 0.0)	0.0 (0.0 - 8.0)	0.0 (0.0 - 0.0)	0.0 (0.0 - 8.0)	0.0 (0.0 - 8.0)	0.247

Data are median (quartile 25% - quartile 75%) or No (%). Percentages may not total 100 because of rounding

CT: computed tomography; PEEP positive end expiratory pressure; ECMO: extracorporeal membrane oxygenation; FiO_2: inspired fraction of oxygen; PEEP: positive end-expiratory pressure; NMBA: neuromuscular blocking agent

* Most recent measurement in 24 hours before intubation, or at ICU admission under invasive ventilation

** Aggregate as the mean of four values

*** Worst value of four available

Table S5 - Clinical Outcomes in the Included Patients According to BMI Category

	Normal (*n* = 244)	Overweight (*n* = 531)	Class I (*n* = 225)	Class II (*n* = 70)	Class III (*n* = 29)	*p* value
**Primary outcome**						
28-day mortality - no. (%)	71 (29.8)	162 (30.9)	51 (23.1)	22 (32.4)	3 (10.3)	0.033
**Secondary outcomes**						
Ventilator-free days at day 28, days	2.0 (0.0 - 18.0)	0.0 (0.0 - 15.0)	5.0 (0.0 - 16.0)	4.5 (0.0 - 17.0)	10.5 (5.0 - 18.0)	0.081
Duration of ventilation, days	13.0 (7.0 - 23.0)	15.0 (8.0 - 24.0)	15.0 (9.0 - 24.0)	12.0 (9.0 - 17.0)	13.0 (7.5 - 21.5)	0.166
In survivors at day 28, days	14.0 (8.0 - 27.0)	16.0 (10.0 - 30.0)	17.0 (10.0 - 28.0)	14.0 (9.0 - 22.8)	15.0 (9.5 - 22.0)	0.204
Tracheostomy – no (%)	47 (19.5)	86 (16.3)	43 (19.2)	7 (10.1)	3 (10.3)	0.286
Thromboembolic complications – no (%)	80 (32.8)	146 (27.5)	62 (27.6)	21 (30.0)	5 (17.2)	0.363
Pulmonary embolism	64 (26.2)	112 (21.1)	55 (24.4)	11 (15.7)	4 (13.8)	0.203
Deep vein thrombosis	12 (4.9)	29 (5.5)	6 (2.7)	7 (10.0)	1 (3.4)	0.151
Ischemic stroke	9 (3.7)	15 (2.8)	2 (0.9)	3 (4.3)	1 (3.4)	0.199
Myocardial infarction	5 (2.0)	9 (1.7)	1 (0.4)	1 (1.4)	0 (0.0)	0.573
Systemic arterial embolism	2 (0.8)	1 (0.2)	0 (0.0)	1 (1.4)	0 (0.0)	0.225
Acute kidney injury – no (%)	104 (42.6)	235 (44.4)	99 (44.4)	36 (51.4)	14 (48.3)	0.755
Need for RRT – no (%)	40 (16.4)	103 (19.4)	37 (16.4)	17 (24.3)	4 (13.8)	0.482
Need of rescue therapy – no (%)*	168 (69.7)	393 (74.7)	162 (73.0)	59 (84.3)	24 (82.8)	0.109
Prone positioning	124 (51.5)	300 (56.9)	131 (58.7)	43 (61.4)	14 (48.3)	0.353
Recruitment maneuver	9 (4.5)	36 (8.2)	12 (6.5)	3 (5.2)	3 (11.1)	0.375
Use of NMBA	95 (38.9)	266 (50.1)	107 (47.6)	42 (60.0)	17 (58.6)	0.006
ECMO	1 (0.4)	6 (1.1)	5 (2.3)	0 (0.0)	0 (0.0)	0.411
ICU length of stay, days	14.0 (8.0 - 26.0)	16.0 (9.0 - 26.0)	16.0 (10.0 - 29.0)	14.0 (10.0 - 21.5)	15.0 (10.0 - 23.0)	0.220
In survivors, days	15.5 (9.3 - 29.0)	18.0 (11.0 - 31.0)	18.0 (11.0 - 30.0)	15.5 (11.0 - 26.5)	15.0 (10.0 - 22.3)	0.376
Hospital length of stay, days	23.0 (13.0 - 37.3)	23.0 (13.0 - 36.0)	26.0 (18.0 - 41.0)	21.0 (12.0 - 31.5)	22.0 (11.5 - 32.0)	0.062
In survivors, days	29.0 (18.0 - 44.5)	30.0 (20.0 - 45.8)	31.0 (22.0 - 44.0)	27.5 (20.8 - 37.3)	24.5 (16.5 - 32.5)	0.195
ICU mortality – no (%)	76 (31.9)	186 (35.8)	59 (27.2)	21 (30.9)	5 (17.9)	0.084
Hospital mortality – no (%)	80 (35.7)	191 (39.0)	60 (29.9)	22 (33.3)	5 (21.7)	0.120
90-day mortality – no (%)	82 (37.4)	201 (40.9)	62 (31.0)	24 (40.7)	5 (19.2)	0.040

Table S5 - Clinical Outcomes in the Included Patients According to BMI Category

	Normal (*n* = 244)	Overweight (*n* = 531)	Class I (*n* = 225)	Class II (*n* = 70)	Class III (*n* = 29)	*p* value

Data are median (quartile 25% - quartile 75%) or No (%). Percentages may not total 100 because of rounding

RRT: renal replacement therapy; NMBA: neuromuscular blocking agent; ECMO: extracorporeal membrane oxygenation; ICU: intensive care unit

* assessed in the first four days of ventilation

Table S6 - Adjusted Effect of Body Mass Index Categories in 28-Day Mortality*

	Overall Group		Mild ARDS		Moderate ARDS		Severe ARDS	
	**Hazard Ratio (95% CI)**	***p* value**	**Hazard Ratio (95% CI)**	***p* value**	**Hazard Ratio (95% CI)**	***p* value**	**Hazard Ratio (95% CI)**	***p* value**
**28-Day mortality**								
Body mass index category								
Normal	1 (Reference)		1 (Reference)		1 (Reference)		1 (Reference)	
Overweight	1.10 (0.83 to 1.47)	0.500	0.69 (0.19 to 2.47)	0.570	1.11 (0.77 to 1.60)	0.570	0.96 (0.53 to 1.72)	0.890
Class I	0.80 (0.55 to 1.17)	0.250	0.12 (0.01 to 1.29)	0.081	0.80 (0.49 to 1.31)	0.380	0.75 (0.38 to 1.52)	0.430
Class II	1.43 (0.87 to 2.36)	0.160	1.08 (0.11 to 10.48)	0.950	0.71 (0.31 to 1.62)	0.420	2.63 (1.16 to 5.97)	0.021
Class III	0.47 (0.14 to 1.51)	0.200	0.00 (0.00 to ∞)	0.999	0.18 (0.02 to 1.35)	0.096	1.83 (0.37 to 9.02)	0.460

Continuous variables were included after standardization and the hazard ratio represents the increase in one standard deviation of the variable.

* All models adjusted for age, hypertension, heart failure, diabetes, chronic obstructive pulmonary disease, use of angiotensin converting enzyme inhibitor, heart rate, mean arterial pressure, pH, PEEP, fluid balance, use of vasopressor and prone positioning on the day of start of ventilation.
